# 127 Ethical Principles of Non-maleficence Care for Burn Patients Under Austere Conditions in the Gaza Strip

**DOI:** 10.1093/jbcr/iraf019.127

**Published:** 2025-04-01

**Authors:** Monica Johnston, Zoe Tao

**Affiliations:** Legacy Oregon Burn Center; Legacy Oregon Burn Center

## Abstract

**Introduction:**

Clinical burn care in the Gaza Strip is burdened by active military destruction of healthcare infrastructure, a large volume of severe burn injuries, and a near-total blockade of resources on the region including crucial medical supplies. With extremely limited resources, burn providers must rapidly make assessments of resource allocation based on initial likelihood of survival while also addressing patient suffering.

**Methods:**

Through the lens of two core ethical principles - justice and non-maleficence - this abstract explores reflections on two clinical cases of critically ill burn patients in the Gaza Strip during a medical mission performed by a U.S. burn nurse with over 20 years of clinical burn care experience.

**Results:**

The first case is a pediatric patient with 90% total burn surface area who died following four days of hospital admission with lack of available wound care, antibiotics, inability to warm the patient, and surgical capabilities. During the entire time, he was in severe pain. The second case is an adult patient with 60% total burn surface area died following four days of hospital admission similarly due to lack of resuscitative fluids and available wound care. On reflection of these two patients’ respective prognoses given austere conditions, precious analgesic resources could have been provided more aggressively during a shortened prognosis and wound care resources potentially allocated to patients with a higher likelihood of survival had these patients been triaged to comfort-focused care upfront.

**Conclusions:**

The practice of clinical burns in conflict zones is burdened by challenging decisions given the limited resources and personnel bandwidth at hand. Ultimately, the end to the siege on hospitals and civilian populations will serve as the most ethical means of burn prevention and resource allocation. While conflict rages on, healthcare workers doing humanitarian work have a moral obligation to exercise principles of distributive justice to balance curative intent treatment and prolonging patient suffering.

**Applicability of Research to Practice:**

This project serves as a primer to introduce the challenging level of adaptability U.S. clinicians traveling to conflict zones face in triaging resuscitative efforts and available analgesia based on extremely limited resources.

**Funding for the Study:**

N/A

